# Rathke’s cleft cysts following transsphenoidal surgery: long-term outcomes and development of an optimal follow-up strategy

**DOI:** 10.1007/s00701-020-04237-5

**Published:** 2020-01-25

**Authors:** Hani J. Marcus, Anouk Borg, Ziad Hussein, Zane Jaunmuktane, Stephanie E. Baldeweg, Joan Grieve, Neil L. Dorward

**Affiliations:** 1 Department of Neurosurgery, National Hospital for Neurology and Neurosurgery, London, UK; 2 Wellcome EPSRC centre for Interventional and Surgical Sciences, University College London, 8.02 Malet Place Building, Gower Street, London, WC1E 6BT UK; 3 Department of Endocrinology, University College London Hospital, London, UK; 4 Department of Neuropathology, National Hospital for Neurology and Neurosurgery, London, UK

**Keywords:** Surgery, Rathke’s cleft cyst, Outcomes, Pituitary

## Abstract

**Background:**

In patients with symptomatic Rathke’s cleft cyst, transsphenoidal surgery is highly effective at preventing further visual loss and usually allows for some recovery of vision. However, cyst recurrence and the need for re-operation are well recognized. To this end, the aim of this study was to investigate patterns of recurrence and long-term outcomes and to use this information to develop an optimal follow-up strategy.

**Method:**

A prospectively maintained database was searched over a 10-year period between 1 January 2008 and the 1 January 2018 to identify all adults that underwent transsphenoidal surgery with a new diagnosis of Rathke’s cleft cyst. A retrospective case note review was performed for each patient to extract data on their presentation, investigation, treatment, and outcome.

**Results:**

In all, 61 eligible patients were identified. The median follow-up was 34 months (range 2–112 months). In the 22 patients with pre-operative visual loss, the outcomes at 6 months were as follows: normal vision (2/22; 9.1%), improved but not normal (7/22; 31.8%), stable (12/22; 54.5%), worse but not blind (1/22; 4.5%), and blind (0/22; 0%). The overall rate of regrowth and re-operation in our study was 19.7 and 11.5%, respectively. The only factor that was significantly associated with recurrence was the presence of residual cystic disease on the post-operative MRI (*p* < 0.001).

**Conclusions:**

We propose a follow-up strategy that stratifies patients at “low risk” if there is no residual cyst, with increasing interval scans, or “high risk” if there is residual cyst, with annual visual assessment and scans.

## Introduction

Rathke’s cleft cysts are benign cystic lesions, believed to be the embryological remnants of Rathke’s pouch, which are located within the sellar and suprasellar region [5, 11]. They are most commonly found incidentally, but may become sufficiently large to compress the optic apparatus and pituitary gland resulting in symptoms such as headache, visual loss, and endocrine dysfunction [7, 19, 26].

The natural history of Rathke’s cleft cysts is highly variable, with some cysts undergoing spontaneous resolution and most remaining stable [2]. In patients with clinical symptoms and radiological progression, treatment is indicated. The gold-standard treatment remains transsphenoidal surgery, and several surgical strategies have been described ranging from simple cyst fenestration to complete resection [20, 23].

Transsphenoidal surgery is highly effective at preventing further visual loss and usually allows for some recovery of vision [19]. However, cyst recurrence and the need for re-operation are well recognized, and patients with treated cysts therefore require long-term follow-up. To this end, the aim of this study was to investigate patterns of recurrence and long-term outcomes following transsphenoidal surgery for Rathke’s cleft cyst and to use this information to develop an optimal follow-up strategy.

## Methods

The Strengthening the Reporting of Observational Studies in Epidemiology (STROBE) Statement was used in the preparation of this section of the manuscript [27].

The study was registered as a service evaluation study with the University College London Hospitals NHS Foundation Trust Clinical Audit Committee. Informed consent was not sought, as this was a retrospective study.

### Setting and participants

The study was conducted at the National Hospital for Neurology and Neurosurgery, which acts as a regional referral centre. During most of the study period, operations were performed by two specialist neurosurgeons (JG and ND), who spent at least half of their clinical programmed activity on pituitary and related tumours.

Each case was discussed in a dedicated weekly multidisciplinary meeting and managed jointly by the surgical and medical team, which includes endocrinologists, ophthalmologists, neuroradiologists and radiation oncologists. Surgery was generally considered indicated where there was clinical and/or radiological compression of the optic apparatus.

All cases were recorded on a prospectively maintained database, and this database was searched over a 10-year period between 1 January 2008 and the 1 January 2018 to identify all adults aged 16 years or more that underwent transsphenoidal surgery with a new diagnosis of Rathke’s cleft cyst.

### Variables and data sources

A retrospective case note review was performed for each patient to extract data on their presentation, investigation, treatment, and outcome.

Data on each patient’s presentation included their age (years), gender (male or female), symptoms and signs. Particular note was made of any visual or endocrine symptoms and signs.

Data on investigation included their endocrine profile and imaging features. Their endocrine profile included levels of prolactin, insulin-like growth factor–1 (IGF-1), morning cortisol, follicle-stimulating hormone (FSH), luteinising hormone (LH), thyroid-stimulating hormone (TSH) and free thyroxine (T4). Patients were considered growth hormone (GH) deficient when low IGF1 was recorded at presentation or there was failure of GH response (< 3μg/L) to provocative pituitary tests such as insulin tolerance test (ITT) and glucagon stimulation test (GST) post-operatively. Adrenocorticotropic hormone (ACTH) deficiency was recorded for patients with low morning cortisol (< 100 nmol/L) or there was failure of cortisol response to ITT and GST post-operatively. Men with low morning testosterone and pre-menopausal women having amenorrhoea or low oestradiol with low or inappropriately normal FSH and LH levels were considered gonadotrophin deficient. In addition, post-menopausal women with low or inappropriately normal FSH and LH were considered deficient. Deficiency of TSH was recorded when TSH was low or inappropriately normal with low free T4 level. Patients on desmopressin replacement were considered to have central diabetes insipidus. Their imaging features were the size, location, and signal characteristics of the Rathke’s cleft cyst on magnetic resonance imaging (MRI).

Data on treatment included the use of a microscope or endoscope, the extent of resection, and the nature of sellar floor reconstruction. The surgical strategy was based on individual surgeon preference, with one surgeon favouring use of a microscope and complete resection where possible (JG), and the other favouring use of an endoscope and simple cyst drainage (ND). In cases where an arachnoid breach was identified, both surgeons reconstructed the sellar floor with an abdominal fat graft, and if the defect was large, a lumbar drain was placed for 3 to 5 days.

Data on pathology findings included the presence of squamous metaplasia and inflammation in the cyst wall.

Data on short-term outcome included length of stay (days) and the presence of complications. Post-operative complications included death, vascular complications, cranial nerve injury, cerebrospinal fluid (CSF) leak, meningitis, visual complications, transient and permanent diabetes insipidus and hypopituitarism. Vascular complications included carotid or other vessel injury or symptomatic haematoma. Venous bleeding from the cavernous sinus was not considered a vascular complication unless it prevented completion of the operation. Epistaxis was not included as a surgical complication unless it required return to operating room. Cerebrospinal fluid leaks included all post-operative leaks.

Data on long-term outcome included their visual outcome, endocrine outcome, and recurrence. Visual outcome and endocrine outcomes were formally assessed at approximately 6 months post-operatively. Visual outcome was recorded as follows: normal, improved but not normal, stable, worse but not blind or blind. Endocrine outcome was based on their baseline endocrine profile and where applicable, dynamic testing (including ITT and FST to assess the GH and ACTH axes). Radiological follow-up was based on individual surgeon preference but usually included an initial scan at 3–6 months, followed by yearly scans for 5 years, and then an increase in the scanning interval. At each point, we recorded whether there was evidence of a residual cyst as reported by a radiologist; if so, whether there was evidence of growth since the last scan; and if so, whether this required re-operation. Our practice is to offer re-operation only if there is clinical and/or radiological compression of compression of the optic apparatus.

### Study size and statistical methods

No formal power calculation was performed. Instead, the sample size was determined on a constraint-based pragmatic approach and on previous cohort studies. We considered a minimum of 50 patients sufficient for meaningful analysis, and it was estimated that this would be achieved over a 10-year period.

Data were analysed using with SPSS v 20.0 (IBM, IL, USA). The mean and standard deviation were calculated for parametric variables, and the median and interquartile ranges were calculated for non-parametric variables. The chi-square test and Fishers exact test were used to compare categorical variables. A value of *p* < 0.05 was considered statistically significant.

## Results

### Presentation

In all, 70 patients were identified that underwent transsphenoidal surgery with a new diagnosis of Rathke’s cleft cyst, of which 61 patients had case notes available. The median age was 55 years (range 16–83 years), and the male:female ratio was 1:2.

The most common symptoms were headache (25/61; 41.0%) and visual loss (22/61; 36.1%). Other symptoms were related to endocrine dysfunction and included lethargy (11/61; 18.0%), sexual dysfunction (7/61; 11.5%), and polydipsia and/or polyuria (3/61; 4.9%). In approximately a quarter of cases (15/61; 24.6%), the cysts were found incidentally.

The most common signs were ophthalmic, included restricted fields (22/61; 36.1%) and reduced visual acuity in one or both eyes (6/61; 9.8%) and optic atrophy (2/61; 3.3%).

### Investigation

Pre-operative endocrine dysfunction related to the anterior pituitary was present in the majority of patients (42/61; 68.9%). The most common deficiencies were as follows: TSH (32/61; 52.5%), adrenocorticotropic hormone (ACTH) (27/61; 44.3%), FSH and LH (29/61; 47.5%), and growth hormone (GH) (16/61; 26.2%). A further four patients had diabetes insipidus (DI) (4/61; 6.6%).

The vast majority of cysts were greater than 10 mm in maximal diameter (54/61; 88.5%) and located within the sellar and suprasellar regions (30/61; 49.2%) or the sellar region alone (25/61; 41.0%); isolated suprasellar cysts were uncommon (6/61; 9.8%). The signal characteristics of cysts on T1-weighted MRI were variable. Cysts were hypointense in 29 cases (29/61; 47.5%), hyperintense in 22 cases (22/61; 36.1%) and isointense in 10 cases (16.4%).

### Treatment

Overall, operations were performed with a microscope in 44 cases (44/61; 72.1%) and an endoscope in 17 cases (17/61; 27.9%). In the majority of cases, a simple cyst fenestration was performed (36/61; 59.0%). Partial resection was performed in 17 cases (17/61; 27.9%), and complete resection was performed in 8 cases (8/61; 13.1%). The sellar floor was reconstructed with a fat graft in approximately half of cases (30/61; 49.2%) and a lumbar drain used in approximately one in ten cases (6/61; 9.8%).

### Outcome

In all cases, the pathological diagnosis was a Rathke’s cleft cyst. Squamous metaplasia was identified in the cyst wall in 9 cases (9/61; 14.8%), and inflammation was identified in 17 cases (17/61; 27.9%).

The median length of stay was 5 days (range 2–29 days). The most common post-operative complication was CSF leak (13/61; 21.3%), and this resulted in meningitis in one case (1/61; 1.6%). In cases of CSF leak, patients were managed with a combination of a fat graft and/or lumbar drainage.

The median follow-up was 34 months (range 2–112 months). All 39 patients with normal vision pre-operatively continued to have normal vision at 6 months from surgery. In the 22 patients with pre-operative visual loss, the outcomes at 6 months were as follows: normal vision (2/22; 9.1%), improved but not normal (7/22; 31.8%), stable (12/22; 54.5%), worse but not blind (1/22; 4.5%) and blind (0/22; 0%).

In the 53 patients in whom complete data were available, endocrine outcomes at last follow-up assessment were as follows: TSH deficiency (36/53; 67.9%), followed by ACTH deficiency (27/53; 50.9%), FSH and LH deficiency (26/53; 49.1%) and GH deficiency (16/53; 30.1%). Overall, there was no significant recovery in the hypothalamic-pituitary-adrenal and thyroid axes post-operatively; 9.4% (5/53) and 11.3% (6/53) developed new ACTH and TSH deficiencies, respectively. However, FSH and LH deficiency, and GH deficiency, were each reversed in 3 cases (3/53; 5.7%). Permanent DI was reported in 14 patients (14/53; 26.4%); 4 patients with pre-operative DI retained the condition and a further 10 patients developed DI post-operatively.

**Fig. 1:**
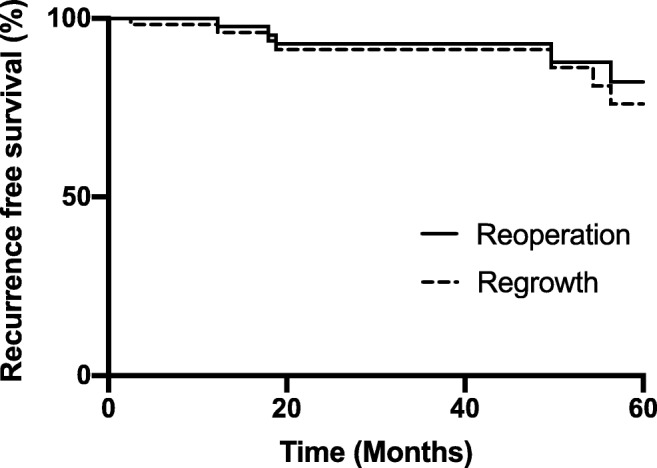
Kaplan-Meier curve illustrating the actuarial recurrence free survival. Time was measured from the initial surgery. Recurrence was determined by post-operative imaging

The actuarial recurrence free survival is illustrated in Fig. 1. The post-operative MRI at 3–6 months demonstrated residual cystic disease in 28 cases (28/61; 45.9%). Of these 28 cases with residual disease, there was a reduction in the cyst size in one case (1/61; 1.6%), stable appearances in 15 cases (15/61; 24.6%) and growth in 12 cases (12/61; 19.7%). Of the 12 cases with growth, this resulted in re-operation in 7 cases (7/61; 11.5%). The median time for re-operation was 50 months (range 12–105 months).

The characteristics associated with recurrence are summarized in Table 1. The only factor that was significantly associated with recurrence was the presence of residual cystic disease on the post-operative MRI at 3–6 months (*p* < 0.001); no patients without residual cystic disease went on to develop later recurrence.

**Table 1:** Characteristics associated with recurrence *A chi-square test and Fishers exact test were used to compare categorical variables against no recurrence, regrowth and re-operation

	No recurrence (%) *n* = 49	Regrowth (%) *n* = 12	Re-operation (%) *n* = 7	*P* value*
Location				0.675
Sellar	22 (44.9)	3 (25.0)	2 (28.6)	
Suprasellar	4 (8.2)	2 (16.7)	1 (14.3)	
Both	23 (46.9)	7 (58.3)	4 (57.1)	
Size				0.803
< 1 cm	5 (10.2)	2 (16.7)	1 (14.3)	
> 1 cm	44 (89.8)	10 (83.3)	6 (85.7)	
Consistency				0.438
Low	24 (49.0)	5 (41.7)	3 (42.9)	
High	16 (32.7)	6 (50.0)	4 (57.1)	
Iso	9 (18.4)	1 (8.3)	0 (0)	
Approach				0.664
Endoscopic	13 (26.5)	4 (33.3)	1 (14.3)	
Microscopic	36 (73.5)	8 (67.7)	6 (85.7)	
Extent of resection				0.865
Fenestration	30 (61.2)	6 (50.0)	3 (42.9)	
Partial resection	13 (26.5)	4 (33.3)	3 (42.9)	
Complete resection	6 (12.2)	2 (16.7)	1 (14.3)	
Sellar floor reconstruction				0.194
Fat graft	22 (44.9)	3 (25.0)	2 (28.6)	
Fat graft and lumbar drain	6 (12.2)	0 (0)	0 (0)	
Neither	21 (42.9)	9 (75.0)	5 (71.4)	
Squamous metaplasia				0.980
Y	7 (14.3)	2 (16.7)	1 (14.3)	
N	42 (85.7)	10 (83.3)	6 (85.7)	
Inflammation				0.896
Y	13 (26.5)	4 (33.3)	2 (28.6)	
N	36 (73.5)	8 (67.7)	5 (71.4)	
Residual cyst				< 0.001
Y	16 (32.7)	12 (100)	7 (100)	
N	33 (67.3)	0 (0)	0 (0)	

The characteristics associated with residual cystic disease are summarized in Table 2. There were no factors significantly associated with residual cystic disease (*p* > 0.05 in all cases).

**Table 2:** Characteristics associated with residual cystic disease on the post-operative MRI *A chi-square test and Fishers exact test were used to compare categorical variables against no residual disease and residual disease

	No residual disease (%) *n* = 33	Residual disease (%) *n* = 28	*P* value*
Location			0.961
Sellar	14 (42.4)	11 (39.3)	
Suprasellar	3 (9.1)	3 (10.7)	
Both	16 (48.5)	14 (50.0)	
Size			0.299
< 1 cm	2 (6.1)	5 (17.9)	
> 1 cm	31 (93.9)	23 (82.1)	
Consistency			0.684
Low	14 (42.4)	15 (53.6)	
High	13 (39.4)	9 (32.1)	
Iso	6 (18.2)	4 (14.3)	
Approach			0.863
Endoscopic	10 (30.3)	7 (25.0)	
Microscopic	23 (69.7)	21 (75.0)	
Extent of resection			0.181
Fenestration	23 (69.7)	13 (46.4)	
Partial resection	7 (21.2)	10 (35.7)	
Complete resection	3 (9.1)	5 (17.9)	
Sellar floor reconstruction			0.401
Fat graft	11 (33.3)	14 (50.0)	
Fat graft and lumbar drain	4 (12.1)	2 (7.1)	
Neither	18 (54.5)	12 (42.9)	
Squamous metaplasia			0.237
Y	7 (21.2)	2 (7.1)	
N	26 (78.8)	26 (92.9)	
Inflammation			0.863
Y	10 (30.3)	7 (25.0)	
N	23 (69.7)	21 (75.0)	
Recurrence			< 0.001
No recurrence	33 (100)	16 (57.1)	
Regrowth	0	12 (42.9)	
Re-operation	0	7 (25.0)	

The outcomes associated with the different surgical strategies are summarized in Table 3. The rates of post-operative CSF leak and recurrence did not differ significantly, regardless of whether the surgeon performed a simple cyst fenestration or attempted complete resection (*p* > 0.05 in all cases).

**Table 3:** Outcomes associated with surgical strategy on an intention-to-treat basis *A chi-square test and Fishers exact test were used to compare categorical variables against fenestration and attempted resection

	Fenestration (%) *n* = 36	Resection (%) *n* = 25	*P* value*
Sellar floor reconstruction			0.391
Fat graft	15 (41.7)	10 (40.0)	
Fat graft and lumbar drain	2 (5.6)	4 (16.0)	
Neither	19 (52.8)	11 (44.0)	
CSF leak			0.920
Y	8 (22.2)	5 (20.0)	
N	28 (77.8)	20 (80.0)	
Residual cyst			0.610
Y	18 (50.0)	10 (40.0)	
N	18 (50.0)	15 (60.0)	
Recurrence			0.557
No recurrence	30 (83.3)	19 (76.0)	
Regrowth	6 (16.7)	6 (24.0)	
Re-operation	3 (8.3)	4 (16.0)	

## Discussion

### Principal findings and proposed follow-up strategy

**Fig. 2:**
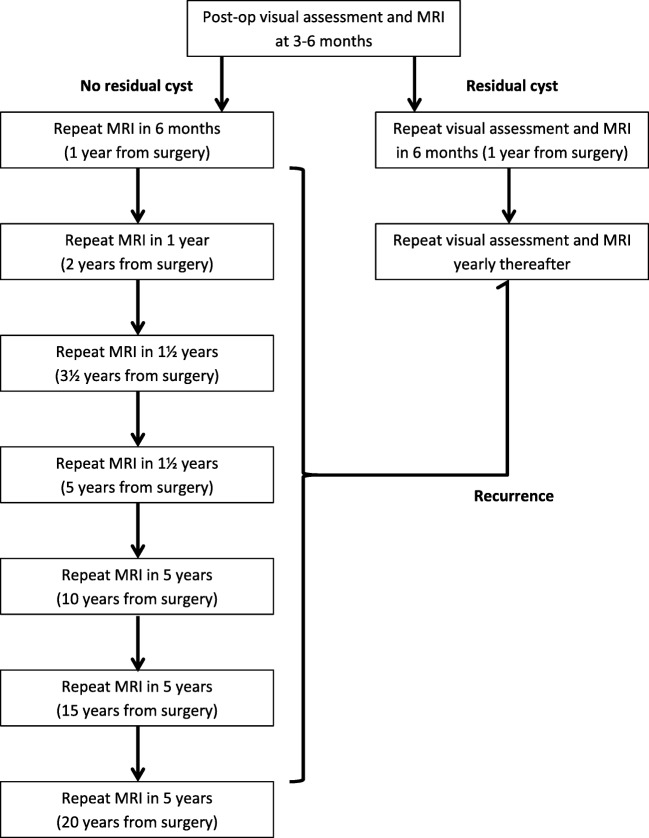
Follow-up strategy for patients with who underwent surgery for Rathke’s cleft cyst

In proposing an optimal follow-up strategy for patients with Rathke’s cleft cysts following transsphenoidal surgery, we considered the following principle findings: (1) residual cystic disease was the only significant risk factor for recurrence; (2) growth kinematics for cysts were variable rather than linear, with spontaneous regression occurring in one cyst and regrowth occurring as late as 10 years from the initial surgery in other cysts and (3) re-operation was required if cysts caused clinical and/or radiological compression of the optic apparatus. With these principles in mind, we propose a follow-up strategy that stratifies patients at “low risk” if there is no residual cyst, with increasing interval scans, or at “high risk” if there is a residual cyst, with annual visual assessment and scans (see Fig. 2).

### Comparison with other studies

In keeping with the literature, the majority of the patients that underwent transsphenoidal surgery for Rathke’s cleft cysts in our cohort presented with headache, visual loss, and endocrine dysfunction [1, 4, 7, 15, 18, 19, 28]. Our finding that pre-operative endocrine dysfunction related to the anterior pituitary was present in over two thirds of patients (42/61; 68.9%) was considerably higher than other groups, with a large meta-analysis reporting a weighted average of 46% [23]. We speculate this may be the result of our joint management of such patients with endocrinologists that are more likely to investigate and report deficiency.

In our cohort, cysts were typically greater than 10 mm in maximal diameter (54/61; 88.5%) and were located within sellar and suprasellar regions or the sellar region alone (55/61; 90.2%). These findings are comparable with the literature, with the aforementioned meta-analysis reporting a weighted average cyst diameter of 15 mm, and a location within the sellar and suprasellar region or the sellar region alone in 94% of cases [23]. Entirely suprasellar Rathke’s cleft cysts are uncommon and, where they occur, are thought to arise from Rathke’s pouch remnants within the pars tuberalis, which lies above the diaphragm [3]. It has been suggested that entirely suprasellar cysts have a greater propensity for recurrence, but we could not find data to support this in our study [7].

The signal characteristics of Rathke’s cleft cysts on T1-weighted MRI were characteristically variable in our cohort; cysts were most commonly hypointense (29/61; 47.5%), but were hyperintense in 22 cases (22/61; 36.1%) and isointense in 10 cases (16.4%). These variable signal characteristics are thought to reflect number and type of secretory cells, the presence or absence of chronic inflammation and the rate of cell desquamation [7, 26]. A number of studies have sought to associate signal characteristics with recurrence but most fail to do so, as is the case with our study [16]. Chotai et al. found that characteristics on T1-weighted MRI were associated with squamous metaplasia but not recurrence and that hypointensity on T2-weighted MRI was associated with recurrence but not squamous metaplasia [7].

Transsphenoidal surgery remains the gold-standard treatment for symptomatic Rathke’s cleft cysts, but a wide range of operative strategies have been described in the literature [1, 4–6, 10, 15, 18–20, 23, 24]. Both microscopic and endoscopic transsphenoidal surgical approaches are reported as being safe and effective options, but the latter has gained popularity in recent years due to technological advances [1, 8, 9, 13, 14, 17, 21, 22, 28–31]. In an meta-analysis of 1151 patients, Mendelson et al. found that the endoscopic approach was associated with a reduced rate of recurrence when compared with the microscopic approach (weighted average of 8 and 14% respectively) [23]. However, the authors acknowledged considerable confounders including the fact that there were far fewer studies reporting the use of an endoscopic approach and that these were more recent.

The optimal extent of surgical resection of Rathke’s cleft cysts is also contentious in the literature; with some surgeons advocating simple cyst fenestration and others complete resection [20, 23]. In a recent meta-analysis of 655 patients, Lu et al. found that complete resection was associated with a reduced rate of recurrence when compared with partial resection or simple fenestration (pooled incidence of 14% and 20% respectively), but also an increased rate of post-operative diabetes insipidus (pooled incidence of 27 and 10% respectively). [20]

We were not able to find data to support a difference in outcomes between the microscopic and endoscopic approach or between simple cyst fenestration and complete resection. However, an important potential confounder in our study was the fact that one surgeon favoured use of a microscope and complete resection where possible and the other favoured the use of an endoscope and simple cyst drainage.

Interestingly, squamous metaplasia and inflammation identified in the cyst wall was an infrequent finding in our study (14.8 and 27.9% respectively) and was not associated with recurrence. This is in contrast to the literature, and Kinoshita et al. suggested that squamous metaplasia may be the most important factor associated with cyst recurrence [16]. The reasons for this discrepancy are unclear and may reflect inconsistent reporting of pathological features.

The rate of post-operative CSF leak in this study (13/61; 21.3%) was rather higher than reported in the literature, and indeed higher than we have found following transsphenoidal surgery for pituitary adenoma (ca. 5%). We speculate that this is because if no obvious CSF leak is identified intra-operatively, we often attempt to establish free drainage of the cyst with the sphenoid sinus. In cases where there is a breach in the arachnoid, either unrecognized intra-operatively or later post-operatively, this can result in a CSF fistula. To address this complication, we have now modified our technique to include an intraoperative Valsalva manoeuvre to check for a CSF leak and a lower threshold for placing an abdominal fat graft and a lumbar drain.

In patients presenting with visual loss, most patients experienced stabilization or improvement of visual symptoms following transsphenoidal surgery for Rathke’s cleft cyst (21/22; 95.4%).

As with other studies, we found that the majority of patients with pre-operative endocrine dysfunction required on-going replacement post-operatively [12, 15]. In particular, patients with ACTH and TSH deficiency, as well as central DI, had no significant improvement post-operatively [25]. It is thought that this limited recovery of endocrine function is due to capsular wall inflammation resulting in chronic hypophysitis or prolonged compression of the gland, leading to irreversible damage.

The rate of new post-operative diabetes insipidus (10/53; 18.9%) was also rather higher than is generally reported in the literature. However, other groups have reported similar findings where resection of the cyst wall is attempted. In a study of 188 patients with Rathke’s cleft cysts treated surgically, Aho et al. found that 17.8% (21/118) exhibited symptoms of diabetes insipidus that had not been present pre-operatively.

The overall rate of regrowth and re-operation in our study was 19.7 and 11.5% respectively. Although these findings are comparable to the literature, few studies explicitly distinguish between radiological regrowth and symptomatic recurrence that requires re-operation [20, 23]. In a recent study of 100 patients undergoing transsphenoidal surgery for Rathke’s cleft cyst, Lin et al. found regrowth in 26.6% of cases and re-operation in 9.2% of cases. As with our study, they found that residual cystic disease on post-operative MRI was most strongly associated with recurrence.

### Limitations

The present study has several limitations. The sample size of 61 patients was small because symptomatic Rathke’s cleft cyst is rare and may have been underpowered to detect more subtle associations. Nonetheless, we met our a priori minimum of 50 patients, and our principle finding that residual cystic disease on post-operative MRI was most strongly associated with recurrence is likely valid.

More generally, although the cases were recorded on a prospectively maintained database, the data was drawn from a retrospective case note review. There was therefore the possibility of incomplete or inaccurate data, selection bias and lack of control.

## Conclusions

In this study, we have, in keeping with literature, confirmed that transsphenoidal surgery is a safe and effective treatment for symptomatic Rathke’s cleft cysts but that there is a risk of recurrence irrespective of the surgical approach selected. To this end, we have identified the most important risk factor for recurrence as the presence of residual cystic disease on post-operative MRI and propose an optimal follow-up strategy based on this finding.
